# Classification models for Invasive Ductal Carcinoma Progression, based on gene expression data-trained supervised machine learning

**DOI:** 10.1038/s41598-020-60740-w

**Published:** 2020-03-05

**Authors:** Shikha Roy, Rakesh Kumar, Vaibhav Mittal, Dinesh Gupta

**Affiliations:** International Centre for Genetic Engineering and Biotechnology, New Delhi, India

**Keywords:** Computational models, Machine learning

## Abstract

Early detection of breast cancer and its correct stage determination are important for prognosis and rendering appropriate personalized clinical treatment to breast cancer patients. However, despite considerable efforts and progress, there is a need to identify the specific genomic factors responsible for, or accompanying Invasive Ductal Carcinoma (IDC) progression stages, which can aid the determination of the correct cancer stages. We have developed two-class machine-learning classification models to differentiate the early and late stages of IDC. The prediction models are trained with RNA-seq gene expression profiles representing different IDC stages of 610 patients, obtained from The Cancer Genome Atlas (TCGA). Different supervised learning algorithms were trained and evaluated with an enriched model learning, facilitated by different feature selection methods. We also developed a machine-learning classifier trained on the same datasets with training sets reduced data corresponding to IDC driver genes. Based on these two classifiers, we have developed a web-server Duct-BRCA-CSP to predict early stage from late stages of IDC based on input RNA-seq gene expression profiles. The analysis conducted by us also enables deeper insights into the stage-dependent molecular events accompanying IDC progression. The server is publicly available at http://bioinfo.icgeb.res.in/duct-BRCA-CSP.

## Introduction

Breast cancer ranks second among all the cancer types arranged in the order of increasing death rates, also the most prevalent cancer in women^1^. The cancer has been categorized into three therapeutic groups: ER - ER+ patients receive endocrine therapy, HER – HER+ group is treated by therapeutic targeting of HER/ERBB2, and TNBC - lacking expression of ER, PR, HER receptors^2^. It has been categorized into two major histological types- Invasive Ductal Carcinoma (IDC) and Invasive Lobular Carcinoma (ILC), occurring in 47–79% and 2–15% of invasive cancers amongst women of different worldwide races, respectively^3,4^. These two sub-types show similarity in certain features such as tumour site, size, stage and grade, but have different metastatic patterns, characteristic histology and malignant calcifications^5,4^. IDC starts from ducts and spreads to the breast fatty tissue, whereas ILC is restricted to milk producing lobules^6^. These two sub-types are also discriminated at the molecular level with differential expression of gene encoding vimentin, cathepsin D, thrombospondin, E-cadherin, vascular endothelial growth factor, cytokeratin 8, and cyclin A^4,7–12^. The pathological differences between the two sub-types arises as a result of separate gene regulatory networks, which warrants further exploration for the development of appropriate diagnostic and therapeutic treatment strategy^6^. According to reports, 75% cases of invasive breast carcinoma cases are accounted by IDC, however, advanced treatment of IDC patients still remains a challenge due to lack of molecular targets for IDC treatment^13,14^. Also, there is the availability of higher number of datasets for IDC patients in TCGA-BRCA, which is favourable for development of efficient classifiers using machine learning. Hence, we implemented machine-learning and developed a web-server for efficient prediction of the correct IDC stage, which can potentially aid in designing appropriate treatment strategies and precise molecular targeting.

Early detection of breast cancer has led to a significant decrease in mortality rate. Prognostic and predictive factors used for therapy are not sufficient and we need new markers for treatment as individuals differ^15^. Although PET and MR imaging techniques are available for early detection of breast cancer, these techniques are based on morphological features that do not provide any clue for molecular events accompanying cancer progression. In these cases, gene expression based analyses are able to capture early stage markers and also detect molecular events and pathways for driving disease from early to late stage. Early availability of such information can lead to identification of patients who would require targeted or personalized therapy. Further, it may also shed some light on tumors for which no standard treatment is available (https://www.cancer.gov/about-cancer/treatment/types/precision-medicine/tumor-dna-sequencing). Unfortunately, imaging techniques will not be help for such treatments. Further, using ML-based methods, not only can the process be automated (thereby eliminating the need of a skilled professional to assess the images), but more information can be derived from a single procedure. Previous studies has been done to identify non coding RNA expression profile associated with early stage of invasive ductal carcinoma^16^. Also early stage markers of breast cancer has been identified using microarray datasets from peripheral blood cell^17^. Machine learning has been previously utilized to discriminate early-late stage based on gene expression profile of clear cell renal cell carcinoma patients^18^. However machine learning based analysis on tissue site based expression profile in invasive ductal carcinoma has not yet been performed.

The increased incidence of breast cancer and higher mortality rate has attracted significant research efforts to unravel its causes, and development of better treatment options^19^. Breast cancer is a heterogeneous disease with varied features, such as morphological appearances, profile, response to therapy, TNM staging, histological grade, etc.^20^. There is a direct correlation between mortality rate and stages of cancer, and the stage progression could be checked by early detection and appropriate treatment strategies^21^. Although knowledge about genomic profiling has been identified in terms of varied molecular features associated with subtypes of cancer, its molecular mechanism of progression is poorly understood^22^. Tumour stage is defined as the anatomic extent of cancer at the time of diagnosis, which is important for an individual patient prognosis, and determination of best treatment strategy^23^. Pierre Denoix and the Union of International Cancer Control (UICC) has classified tumour staging based on TNM classification^23^. TNM classification overlaps with breast cancer stages, where T describes the extent of a primary tumour by the size or depth of invasion mainly in stage I or II, N describes the extent of regional lymph node metastasis in mainly stage II or III, and M describes the presence of metastasis mainly in stage IV^23^. The incorporation of this staging system into molecular or genetic profiles can help in detecting prognostic groups that guide the disease intervention^23^. There is a sharp decrease in the 5-year survival rate of patients with the stage-wise progression of breast cancer^21^. Treatment of cancer remains a challenge because of the lack of knowledge about factors for cancer progression and metastasis^23^. Potential treatment options are available based on clinical and pathological prognostic factors with the histological grade being the most important predictive factor^23^. High throughput techniques such as Next Generation Sequencing (NGS) that capture expression of thousands of genes in a single assay can act as powerful analytical tools for capturing breast cancer prognostic signature^20^. We can obtain information about a large number of genes, but their intertwining relationship cannot be captured by traditional techniques like statistical and correlational analyses, hence advanced methods such as machine-learning are important to capture cryptic signatures inherent in these data^19^. Molecular profiling helps in finding predictive information and identifying prognostic biomarkers that can serve as therapeutic targets^20^. Most of the cancer research is focussed to determine for finding driver genes, which are related to chimeras or splice junctions, which do not utilize the high resolution features of RNA-seq^24^. Machine-learning techniques are increasingly being used for modelling the progression and treatment of cancer due to its ability to detect key features from complex datasets^25^. Personalized treatment strategies could be developed for patients with similar molecular sub-types based on the patterns identified from systematically collected molecular profiles of tumour samples^26^. In this study, we developed classification methods to analyse the genomic datasets of invasive ductal carcinoma obtained from TCGA, using supervised machine-learning algorithms and feature selection methods. We developed prediction models that could discriminate between early and late stages of IDC using RNA-seq datasets. Different feature selection methods such as RFE, RLASSO, linear modelling, linear regression and random forest were trained and evaluated using Python scikit-learn library which provides individual rankings to gene features. Based on the most comprehensive ranking of gene features by various feature selection methods the top gene features were selected for enriched classifier training that helped us efficiently classify the tumours based on the tumour stage-specific gene expression profiles.

## Results

**Figure 1:**
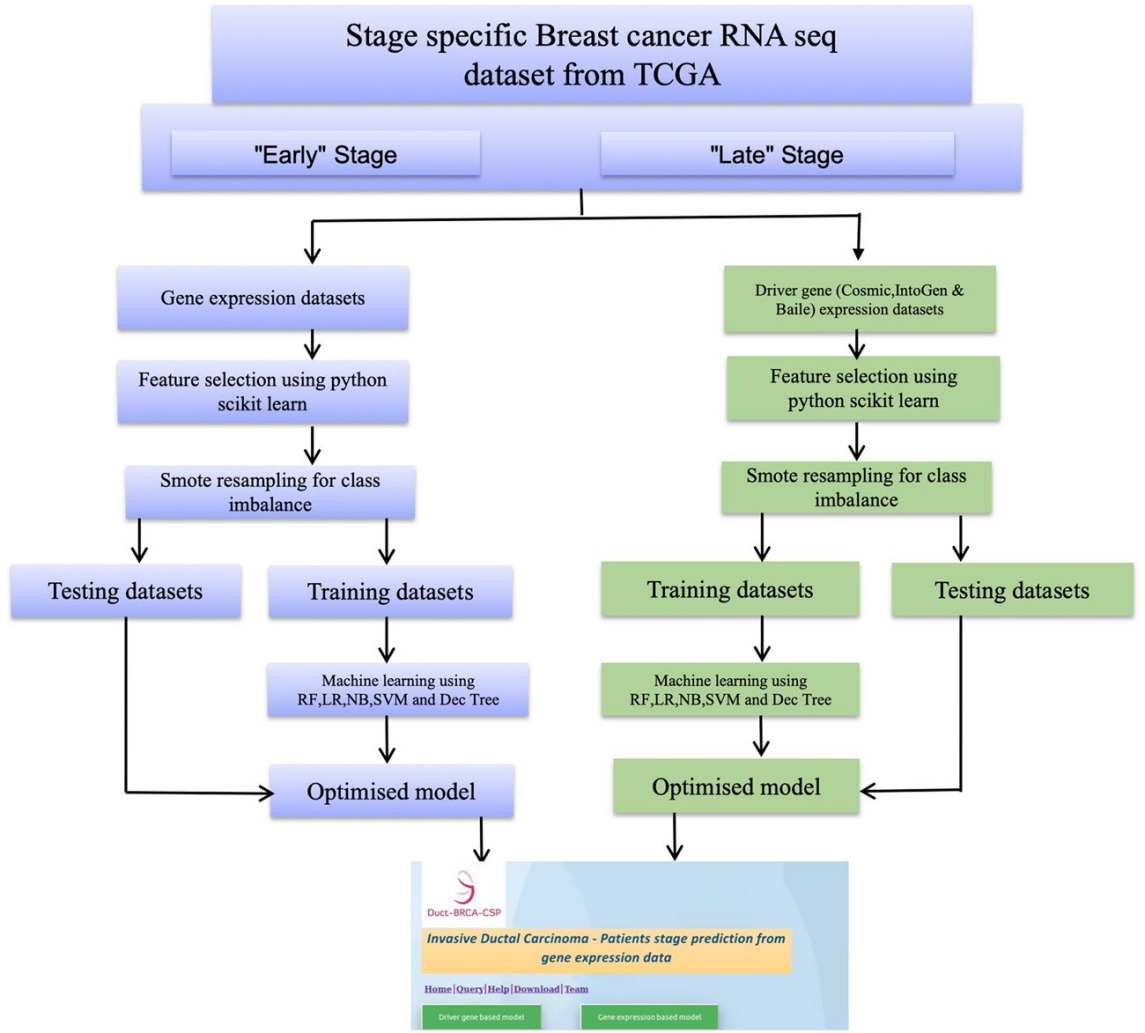
Flowchart of the study to develop classification models, trained with relevant gene expression profiles to efficiently discriminate between the early and late IDC stages.

The workflow followed in our study is shown in Fig. 1. The TCGA level 3 RNA-seq datasets representing 1,093 breast cancer patients were retrieved using the TCGA2STAT R package^27^. The datasets represent 610 IDC patients, the distribution of samples across testing and training set by tumour stage is given in the Table 1. TCGA2STAT package merges the molecular profile information with clinical information into a data frame that is ready for supervised machine-learning. Each of the molecular profiles consists of RNA-seq gene expression data of 20,505 genes. The import dataset consists of ‘expression’ representing the gene expression profiles of patients in terms of RPKM values (described in methods), ‘clinical data’ which consists of clinical information related to patients, and ‘merged data’ in which both the information is mapped. Samples without clinical stage assignments were excluded from our study. Samples bearing clinical stages of stage I and II were pooled together as ‘early stage’, while the stages III and IV were pooled together as ‘late stage’. We generated gene expression data frames as comma separated value (CSV) format from the data retrieved using TCGA2STAT R package, with 20505 genes as column labels and 610 TCGA patient IDs as row labels. The values obtained by mapping the reads to genome generated as gene expression estimates were used as feature vectors for training the machine-learning classifier. Hence, the entire dataset consists of a gene expression data frame with a dimension of 610 * 20505. Near zero variance features and features having correlation coefficient more than 80% were removed using caret, an R package^28^. This led to a preliminary reduction of the number of features from 20,505 to 17,373. The training datasets were standardized using z-score normalization. It converts all the features to common scale with mean zero and standard deviation 1 (Supplementary file, Figure S1, S2). The normalized data-set was used for model generation to discriminate early versus late stages of the cancer.

**Table 1:** Summary of the training and testing data-sets for each stage.

Class label	Clinical status	Samples	Testing	Total
Early stage	Stage I	85	22	107
Early stage	Stage II	290	72	362
Late stage	Stage III	102	26	128
Late stage	Stage IV	10	3	13
**Total**		**487**	**123**	**610**

The normalized datasets were divided into two training sets, the first dataset comprises of complete gene expression datasets which were the original datasets representing expression of 17,373 genes used for feature selection. The second dataset consists of gene expression data corresponding to the driver-gene list in which the training genes were reduced to driver-genes responsible for progression of different cancers. The list of 881 driver genes was obtained from three well curated driver genes lists- Cosmic, IntoGen and Bailey^29–31^. The gene expression of the selected genes of the two datasets were further used for feature selection and classifier model generation (for details, see methods section).

**Figure 2:**
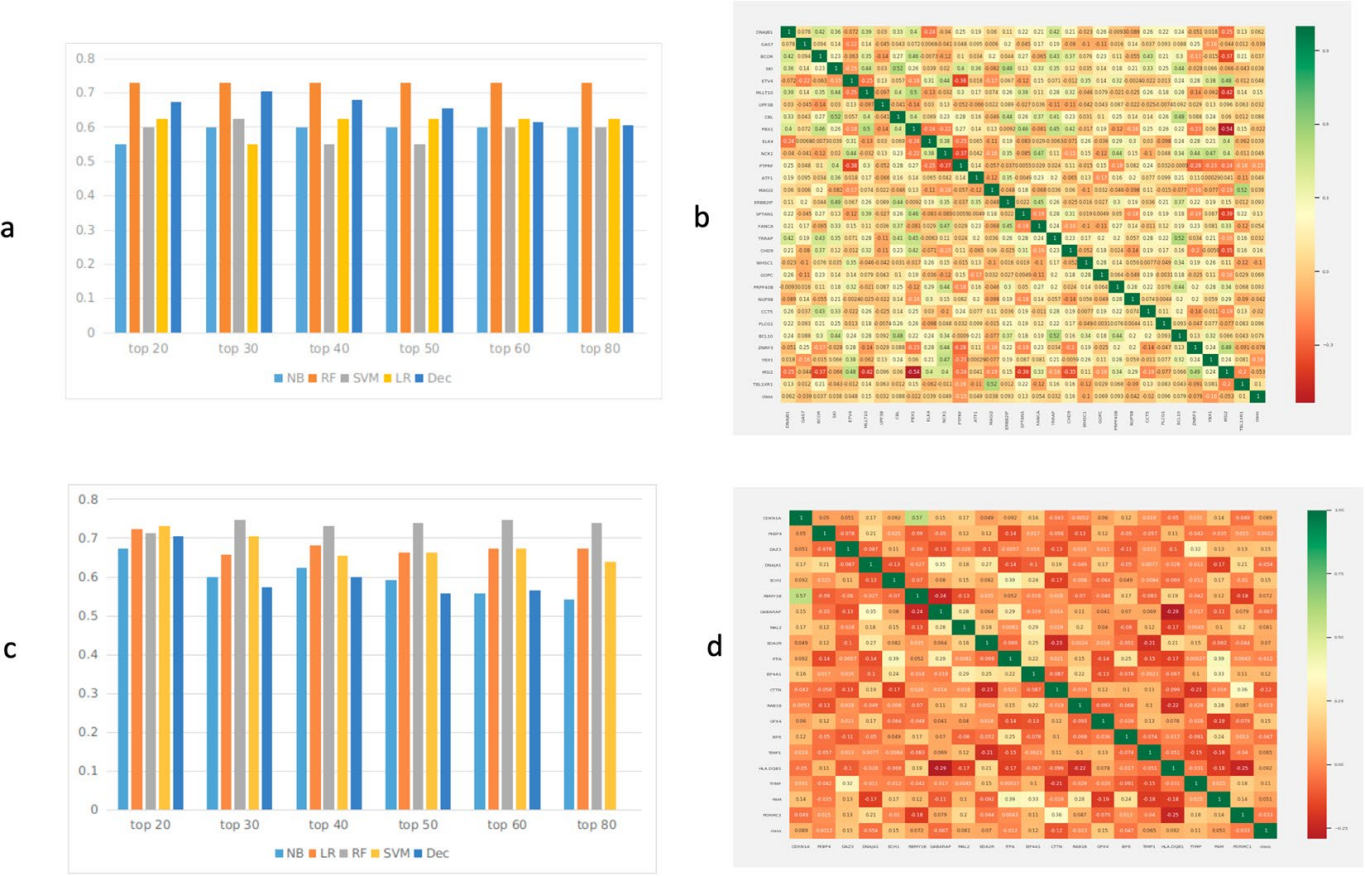
(**a**) Feature selection methods were used to rank the gene features used in the training datasets. Top 20, 30, 40, 50, 60 and 80 features were used to train the binary classification model and their accuracy was evaluated. Based on that, top 30 gene features renders highest accuracy for all the machine learning algorithms evaluated by us. NB: Naïve Bayes, LR: Logistic Regression, RF: Random Forest, SVM: Support Vector Machine, DT: Decision Tree. X- axis: model accuracy, Y-axis: no. of features selected for model building (**b**). Correlation plot for top 30 gene features used in classification model building. X axis: Genes selected by feature selection Y axis: Genes selected by feature selection. (**c**) Feature selection methods were used to rank the gene features used in the training datasets. Top 20, 30, 40, 50, 60 and 80 features were used to train the binary classification model and their accuracy was evaluated. Based on that, top 20 gene features renders highest accuracy for all machine learning algorithms. NB: Naïve Bayes, LR: Logistic Regression, RF: Random Forest, SVM: Support Vector Machine, DT: Decision Tree. X- axis: model accuracy Y-axis: no. of features selected for model building (**d**). Correlation plot for top 20 driver gene features used in model building X, Y axis: Genes selected by feature selection.

The top 30 gene feature list enriched models rendered the highest accuracy for driver gene expression with a mean accuracy of 0.64 for all the machine-learning methods, hence, these features were used for training the model (Fig. 2a,b). The relevance of selected gene features was further validated by survival Kaplan-Meir estimate. Survival estimate revealed that median survival in cases with alteration 95.63 months and cases without alteration 129.6 months (Supplementary file, Figure S7). Top 20 gene feature enriched models gave the highest accuracy for the complete gene expression-based model with a mean accuracy of 0.70 for all the machine-learning methods hence, these features were used for training the models (Fig. 2c,d). The relevance of selected gene feature was further validated by survival Kaplan-Meir estimate. Survival estimate revealed that median survival in cases with alteration months 128.98 months and cases without alteration 129.6 months (Supplementary file, Figure S8). We also performed gene ontology enrichment analysis of selected gene features in biological process of cancer for both the models (Supplementary file, Table ST1). Despite using relevant features, the accuracy was low as the dataset was not balanced, i.e., there are more samples representing early stage as compared to that of late stage (469 for early stage, 141 for late stage). In order to tackle the class imbalance, Synthetic Minority Oversampling Technique (SMOTE) was employed using Python scikit-learn library. SMOTE was employed using ENN (Edited Nearest Neighbour) in which oversampling and under-sampling is performed until there is no difference with k- neighbour of majority class^32^. Real world datasets have higher composition of ‘normal class’ as compared to ‘abnormal class’, introducing bias in classification model. Combination of over-sampling of minority class along with under-sampling of majority class can aid in increasing the classifier performance^33^. To check the SMOTE resampling, models were trained on datasets where SMOTE resampling was employed (Supplementary file, Figure S5). The dataset where SMOTE was employed, the classification accuracy improved from 77% to nearly 89% on the validation set (Supplementary file, Figure S6, S11, S12). For training, validation, and testing, the samples were randomly stratified and split into 80% training-cum-validation sets (available on duct-BRCA-CSP webserver) and 20% independent testing datasets (available on duct-BRCA-CSP webserver).

### Training-cum-validation

**Figure 3:**
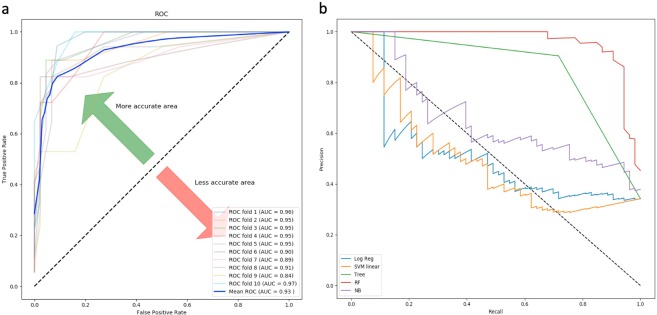
(**a**) Random forest based model achieved maximum performance with auROC of 0.93 on the training dataset when evaluated using ten-fold cross-validation, for the complete gene expression-based model. (**b**) Precision-recall curve is a trade of between precision and recall with high area under the curve representing low false positive and low false negative for all classifiers. Amongst all the prediction models, Random forest achieved the maximum area under precision-recall curve for complete-gene expression model.

**Figure 4:**
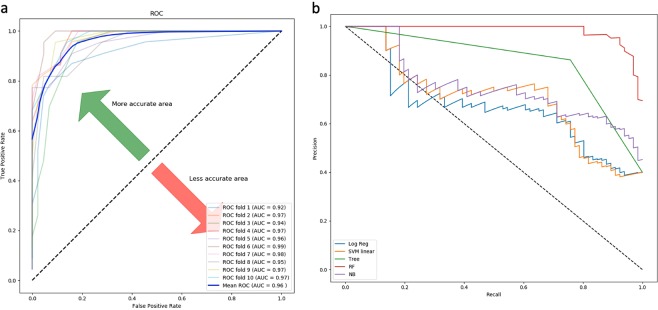
(**a**) Random forest based model achieved maximum performance with auROC of 0.96 on the training dataset when evaluated using ten-fold cross-validation, for the driver gene expression-based model. (**b**) Precision-recall curve is a trade of between precision and recall with high area under the curve representing low false positive and low false negative for all classifiers. Amongst all the prediction models, the Random forest model achieved the maximum area under precision-recall curve for driver-gene expression-model.

The classification accuracy of the generated prediction models ranges from 74% for SVM, to 95% for Random forest; and auROC value ranges from 0.76 for LR to 0.93 for the Random forest trained model for complete gene expression-based model. Based on the model accuracy and auROC, we inferred that the Random forest based prediction model has outperformed the other four machine-learning algorithms implemented in the study (Table 2). Random forest based model achieved the best performance with auROC of 0.93 on the training dataset, evaluated using ten-fold cross-validation for the complete gene expression-based model (Fig. 3a). The Random forest model displayed highest auROC as compared to the other models for complete gene expression-based model(Fig. 3b). The classification accuracy of the generated prediction models ranges from 72% for NB, to 92% for Random forest; and auROC value ranges from 0.72 for LR to 0.96 for Random forest for driver gene expression-based model. Based on accuracy and auROC, we inferred that Random forest based prediction model has outperformed the four other machine-learning algorithms implemented in the study. (Table 2). Random forest based model achieved maximum performance with auROC of 0.96 on training dataset when evaluated using ten-fold cross-validation for driver gene expression-based model (Fig. 4a). Random forest model exhibited the highest area under the curve as compared to the other models for driver gene expression-based model (Fig. 4b).

**Table 2:** Performance of prediction model generated by tenfold cross validation on training cum validation datasets.

Training set	Model	ACC	SEN	SPC	MCC	auROC
Complete Gene expression	RF	95	96	92	0.86	0.93
	DT	85	86	79	0.61	0.77
	NB	75	79	60	0.35	0.77
	LR	74	74	66	0.23	0.76
	SVM	74	73	95	0.29	0.80
Driver gene expression	RF	92	90	98	0.82	0.96
	DT	82	84	77	0.60	0.80
	NB	72	75	63	0.34	0.75
	LR	73	74	66	0.35	0.72
	SVM	76	76	76	0.43	0.76

Accuracy (ACC), sensitivity (SEN) and specificity (SPC) values in %.

### Independent data-set performance

Further, we evaluated the performance of the trained models on independent datasets. The performance was re-evaluated based on accuracy, sensitivity, specificity, MCC and auROC for all the models. We observed coherence in the performance of the models between independent data testing and 10-fold cross validation based on auROC values for the complete gene expression-based model. Random forest achieved maximum auROC of 0.96 with an accuracy of 90% for testing datasets implemented in the complete gene expression-based model (Table 3, Supplementary file, Figure S9). Also, we observed coherence in the performance of the models between independent data testing and 10-fold cross validation based on auROC values for driver gene expression-based model. Random forest achieved maximum auROC of 0.99 with an accuracy of 94% for testing datasets in driver gene expression-based model (Table 3, Supplementary file, Figure S10).

**Table 3:** Performance of prediction models using standard statistical evaluation parameters for independent testing dataset.

Training set	Model	ACC	SEN	SPC	MCC	auROC
Complete Gene expression	RF	90	89	91	0.78	0.96
	DT	84	84	84	0.65	0.81
	NB	71	73	65	0.35	0.82
	LR	67	67	71	0.23	0.62
	SVM	74	72	1	0.36	0.57
Driver gene expression	RF	94	94	1	0.88	0.99
	DT	84	84	83	0.65	0.81
	NB	73	75	71	0.42	0.82
	LR	74	74	76	0.43	0.75
	SVM	77	75	87	0.51	0.73

Accuracy(ACC), sensitivity(SEN) and specificity(SPC) values in %.

### External validation for a microarray dataset

We also evaluated the performance of the models developed by us for another dataset representing a microarray data, obtained from GEO. The models were able to achieve a maximum auROC of 0.47 with an accuracy of 67% for the Random forest based model (Table 4). A maximum auROC of 0.45 with accuracy 38% with Random forest based model trained on driver gene expression features (Table 4). Heatmap of differential expression analysis of microarray datasets between early and late stage for the complete gene expression-based features set (Supplementary file, Figure S3); and driver gene-based features set, showing differences in gene expression between early and late stages for the selected gene features (Supplementary file, Figure S4).

**Table 4:** Performance of prediction models using standard statistical evaluation parameters for external validation dataset.

Training set	Model	ACC	SEN	SPC	MCC	auROC
Complete Gene expression	RF	67	68	50	0.07	0.47
	DT	54	64	23	−0.11	0.44
	NB	70	69	1	0.27	0.60
	LR	63	68	37	0.04	0.53
	SVM	67	67	0	0	0.57
Driver gene expression	RF	38	57	26	−0.16	0.45
	DT	34	1	33	0.09	0.51
	NB	36	75	33	0.04	0.51
	LR	36	54	24	−0.22	0.44
	SVM	38	57	26	−0.16	0.45

Accuracy (ACC), sensitivity (SEN) and specificity (SPC) values in %.

### t-SNE (T-distributed stochastic neighbour embedding)

**Figure 5:**
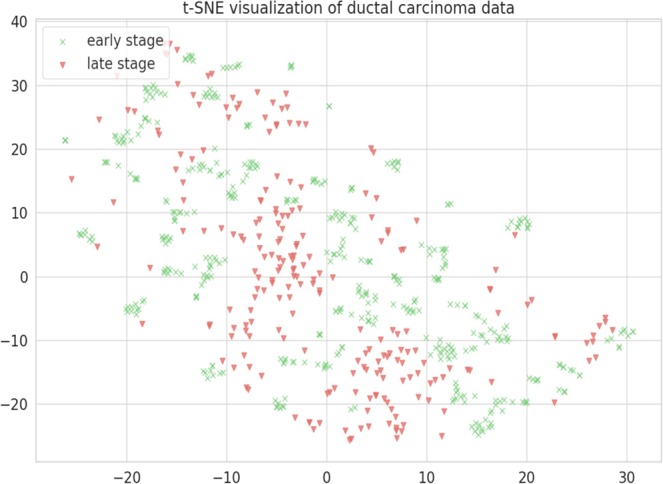
t-SNE visualization was implemented on our gene expression data-sets to check if data-sets are segregating to early stage and late stage class labels based on selected features. This technique visualize our data-sets in 3D space in which early stage and late stage samples are segregating. X axis: X in t-SNE Y axis: Y in t-SNE.

t-SNE technique was used for visualization of our gene expression datasets that displays high-dimensional data providing each data point a location in 2D or 3D space. It helps to model features into high-dimensional object to three-dimensional space such that similar objects tend to cluster together and dissimilar ones are modelled to distant points. The t-SNE analysis on our datasets segregates samples representing early and late stages, which shows that the dataset features are separable (Fig. 5).

### Protein-protein interaction analysis of genes selected for model building

**Figure 6:**
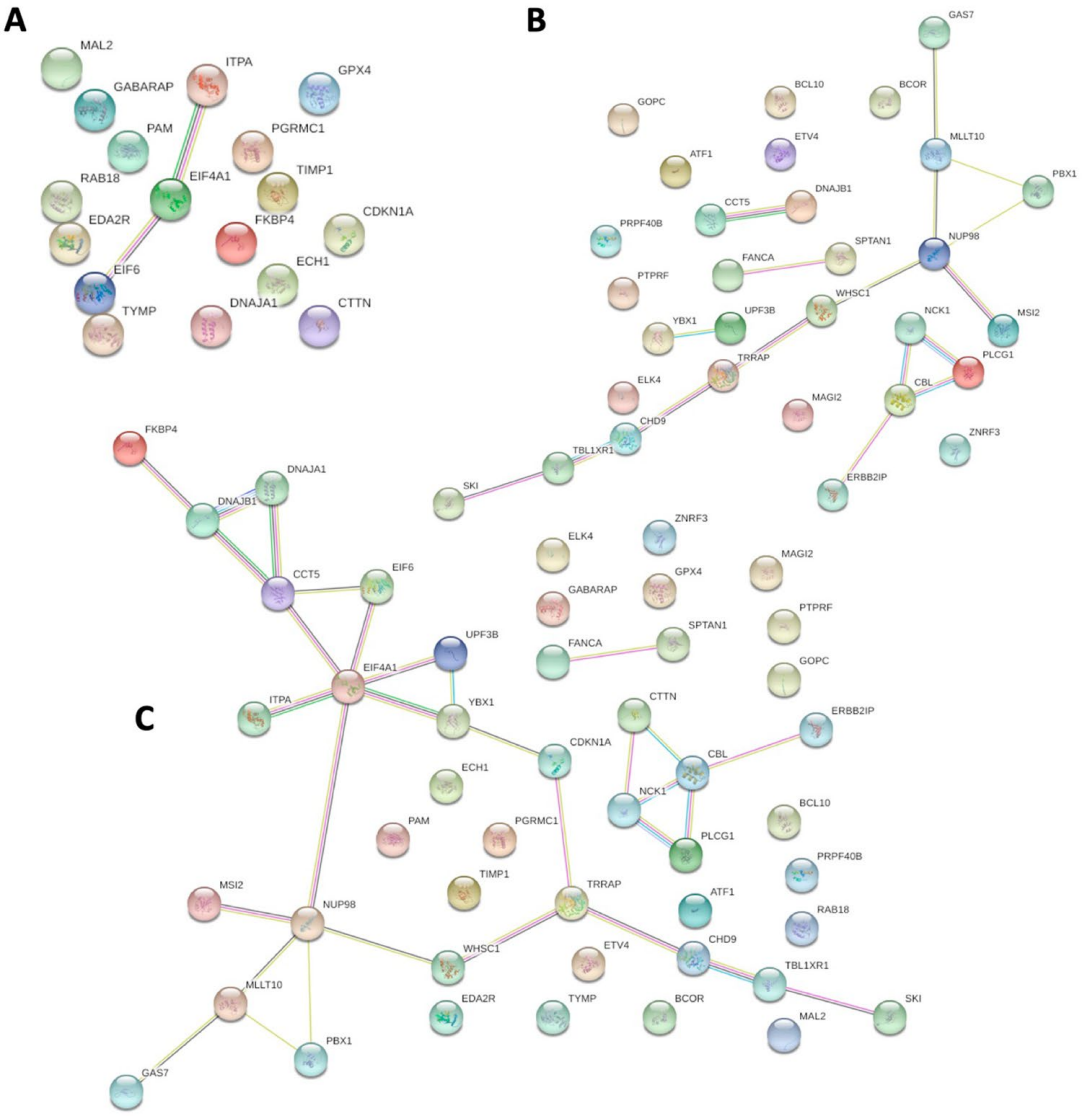
(**a**) Protein-protein interaction analysis using STRING of the gene set for the complete gene expression based-model. (**b**) STRING protein-protein interaction analysis of gene set from driver gene expression-based model. (**c**) STRING protein-protein interaction analysis of the combined gene sets. We found that as compared to the 11a and 11b, more interacting partners are exhibited by STRING analysis of their combination 11c which helps to decipher major pathways associated with IDC progression.

We performed protein-protein interaction analysis on gene features selected by our models using STRING database (Search Tool for Retrieval of Interacting Genes): the complete gene expression-based model, driver gene-based model and the combination of two. We found that as compared to the former two gene sets, more interacting partners are exhibited by STRING analysis of their combination (Fig. 6a-c). Thus, we were able to decipher major pathway that were targeted by gene sets in IDC selected by our models.

Several of these genes have been suggested to play a role in tumor progression from early to late stage. Genomic instability or DNA damage repair is the main driving factor of early stage of cancer development^34^. Cell adhesion and ECM pathway interaction are found to be dys-regulated in early tumorigenesis of ER+ cancer^35^. Whereas in later stages, patients diagnosed at stage IV, develop distant metastasis, which becomes nearly incurable. Although strategies targeting primary tumor has improved, treatment strategies for preventing metastasis is less developed which may be catered using machine learning^36^. Advanced stage of breast cancer is accompanied by genetic marker associated by cell division and proliferation pathway (https://ww5.komen.org/BreastCancer/RecommendedTreatmentsforMetastaticBreastCancer.html). In our analysis we have discovered several genes associated with these pathways suggesting their role in progression from early stage to late stage.

Four proteins encoded by DNAJB1, DNAJA1, CCT5 and FKBP4 are revealed to be in direct interactions, using STRING analysis. These proteins are major components of ubiquitin protein conjugation pathway by interacting with heat shock protein (Fig. 6c). This process mediate cellular processes such as protein localization, cell cycle regulation and DNA damage repair^37^. Ubiquitin dys-regulation can affect tumour suppressor or oncogene leading to cellular transformation and cancer^38^. DNAJB1 binds to mitogen-inducible gene MIG6, a tumour suppressor, which positively regulates epidermal growth factor signalling, leading to breast cancer development^39^.

CCT5 belongs to CCT gene family that serves as potential biomarker and display alteration in majority of breast cancer cases^40^. FKBP4 is found to be over-expressed in ductal carcinoma and under-expressed in lobular carcinoma by expression profiling^41^.

Five proteins encoded by CTTN, NCK1, CBL, PLCG1 and ERBB2IP depicts direct interaction in STRING analysis involved in RTK signalling pathway (Fig. 6c). Its aberrant expression results in enhanced cell proliferation, survival and metastasis leading to malignancy^42^. CTTN encodes cortactin which is a substrate for tyrosine Src nonreceptor tyrosine kinase whose amplification has been reported in primary metastatic breast carcinoma^43^.

NCK1 is an tyrosine kinase that regulation cell adhesion and has role in breast carcinoma cell invasion and metastasis^44^. CBL over-expression results in inhibition of transforming growth factor tumor suppressor activity and breast cancer prognosis^45^. PLCG1 is differentially regulated in breast cancer and has role in tumorigenesis of mediating intercellular signalling cascade^46^. ERBB2IP is a tyrosine kinase that interact with chaperon protein HSP90 and regulates breast tumor progression^47^.

Four proteins encoded by TRAAP, CDKN1A, CHD9 and WHSC1 depict direct interaction in STRING analysis involved DNA replication and DNA damage repair pathway (Fig. 6c). TRAP bind to proliferating cell nuclear antigen (PCNA) resulting DNA replication inhibition and cell growth inhibition and cancer^48^. WHSC1 is a methyl transferase that performs histone methylation affecting cell ability to undergo DNA damage repair^49^.

CDKN1A is a candidate breast cancer biomarker with upregulated expression in breast cancer tissue as compared to adjacent non-tumorous breast tissue^50^. CHD9 is a chromodomain helicase, found to be under-expressed in tumor with high Nottingham prognostic index (NPI) widely used for breast cancer prognostic in METABARIC cohort^51^.

Five proteins encoded by EIF6, ITPA, YBX1, UPF3B and EIF4A1 depict direct interaction in STRING analysis involved in protein translational machinery (Fig. 6c). Deregulated protein synthesis can affect several processes such as cell growth, proliferation, apoptosis at translational level and malignancy^52^. Dys-regulation of EIF4A1 protein results in preferred translation of gene involved in pro-oncogenic signalling^53^.

EIF6 is potential biomarker for cancer as it downstream modulator of cell division resulting in oncogenesis^54^. ITPA downregulation promotes DNA instability, suppression of cell growth and apoptosis in SKBR3 cell lines^55^. YBX1 is an oncoprotein that regulates tumorigenesis and malignant progression in breast cancer^56^. UPF3B is prolactin induced protein that regulates cell cycle progression and found to be upregulated in majority of breast cancer^57^.

Proteins encoded by GAS7, NUP98, MSI2, MLLT10 and PBX1 depict direct interaction in STRING analysis involved dys-regulated DNA binding transcription factor pathway (Fig. 6c). DNA binding TFs are commonly deregulated in cancer which modulates gene expression resulting IN malignancy^58^. MSI2 directly regulates estrogen receptor by binding to ESR1 resulting in breast cancer cell growth^59^.

MLLT10 is one of the breast cancer susceptibility loci identified by genome wide association studies^60^. NUP98 overexpression correlates with poor outcome in breast cancer^61^. GAS7 expression negatively correlates with p53 expression that results in early onset of breast cancer^62^. PBX1 is found to be up-regulated in metastatic progression ERα-positive breast cancer^63^.

### Threshold value of expression for genes selected by feature selection

Threshold value is the expression value beyond which the sample will segregate into two groups, in our study- ‘early’ and ‘late’ stages. For example, if Z-score of CDKN1A (over-expressed in early stage) is greater than 0.32 is then it is representative of an early stage sample otherwise if it is less than 0.32 then it is representative of a late stage sample. We calculated threshold for all the genes selected by feature selection methods for the complete gene expression-based model as well as driver gene-based model (Table 5).

**Table 5:** Threshold value between early-late segregation for genes selected by the models.

Training set	Gene	Threshold	ROC	Differential expression
Complete Gene expression	CDKNIA	0.32	0.56	Upregulated
	FKBP4	0.26	0.509	Upregulated
	DAZ3	0.36	0.556	Upregulated
	DNAJA1	0.28	0.501	Downregulated
	ECH1	0.28	0.576	Upregulated
	RBMY1B	0.35	0.515	Upregulated
	GABARAP	0.32	0.515	Downregulated
	MAL2	0.31	0.611	Upregulated
	EDA2R	0.31	0.579	Upregulated
	ITPA	0.34	0.53	Upregulated
	EIF4A1	0.3	0.629	Upregulated
	CTTN	0.27	0.523	Upregulated
	RAB18	0.24	0.518	Upregulated
	GPX4	0.45	0.535	Upregulated
	EIF6	0.3	0.569	Upregulated
	TIMP1	0.34	0.572	Downregulated
	HLA-DQB1	0.33	0.566	Downregulated
	TYMP	0.345	0.571	Upregulated
	PAM	0.28	0.517	Downregulated
	PGRMC1	—	—	Downregulated
Driver gene expression	DNAJB1	0.29	0.519	Upregulated
	GAS7	—	—	Downregulated
	BCOR	0.34	0.636	Upregulated
	SKI	0.25	0.573	Downregulated
	ETV4	0.257	0.556	Downregulated
	MLLT10	0.32	0.582	Upregulated
	UPF3B	0.36	0.513	Downregulated
	CBL	0.33	0.558	Downregulated
	PBX1	0.27	0.602	Upregulated
	ELK4	0.27	0.601	Upregulated
	NCK1	0.356	0.578	Downregulated
	PTPRF	0.306	0.519	Downregulated
	ATF1	0.32	0.558	Downregulated
	MAGI2	0.27	0.61	Downregulated
	ERBB2IP	—	—	Upregulated
	SPTAN	0.29	0.509	Downregulated
	FANCA	0.31	0.58	Downregulated
	TRRAP	0.27	0.613	Downregulated
	CHD9	0.31	0.579	Downregulated
	WHSC1	—	—	Upregulated
	GOPC	—	—	Downregulated
	PRPF40B	0.33	0.625	Upregulated
	PLCG1	0.33	0.582	Upregulated
	BCL10	0.36	0.539	Downregulated
	NUP98	0.263	0.628	Downregulated
	ZNRF3	—	—	Downregulated
	YBX1	0.41	0.509	Downregulated
	MSI2	—	—	Upregulated
	TBL1XR1	0.41	0.511	Upregulated
	CCT5	0.37	0.567	Upregulated

### Gene ontology

**Figure 7:**
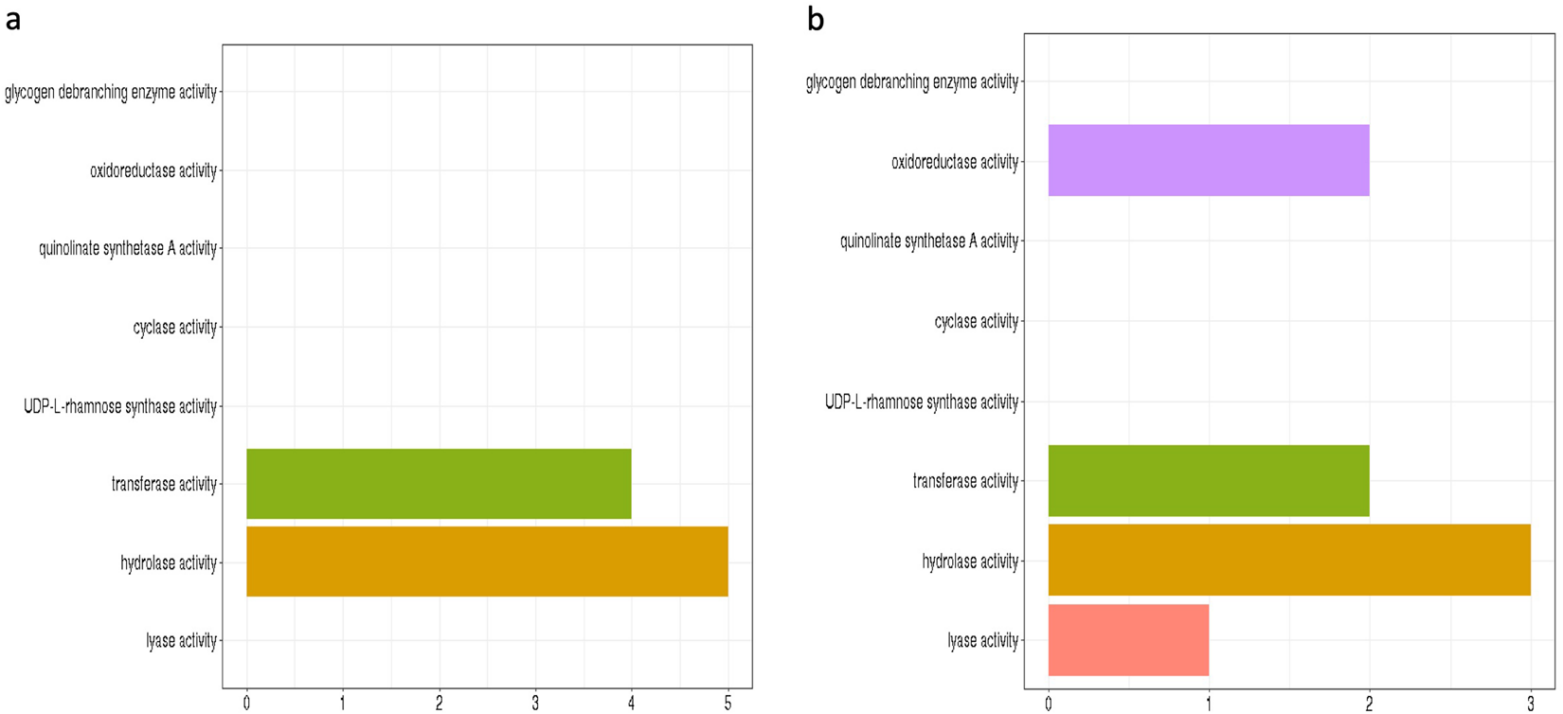
(**a**) Gene ontology analysis of the gene set from driver gene expression-based model for molecular function. (**b**) Gene ontology analysis of gene set from complete gene expression-based model for molecular function.

Clusterprofiler R package was used for gene ontology enrichment analysis of the gene set selected for the complete gene expression-based model and selected gene set for the driver gene-based model. It reveals enrichment in molecular functions such as transferase and hydrolase activity for gene set for the driver gene-based model (Fig. 7a). Cathepsin D is a lysosomal hydrolase which is having increased expression in tumors that results in degradation of extracellular matrix causing metastasis^64^. Increased expression of glycoprotein-sialytransferase is associated with altered membrane synthesis resulting in invasiveness and neoplastic state^65^.

The selected training gene set for the complete gene expression-based model was found to be enriched in molecular functions related to oxidoreductase activity, lyase, hydrolase and transferase activity (Fig. 7b). Glutathione-dependent oxidoreductase- CLIC3 is secreted by cancer cell which contributes to tumour micro-environment by promoting angiogenesis and tumour cell invasion^66^. CSE (Cystathion-gamma-lyase) regulates STAT3 signalling which promotes cell proliferation in breast cancer^67^.

**Figure 8:**
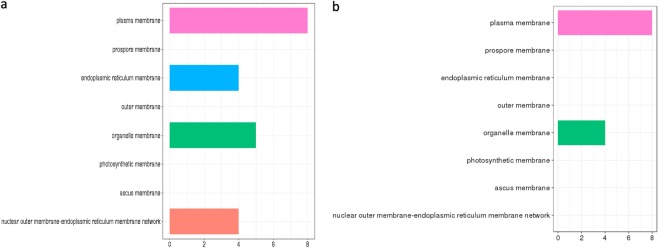
(**a**) Gene ontology analysis of gene set from feature selection from complete gene expression-based model for cellular component. (**b**) Gene ontology analysis of gene set by from driver gene expression-based model for cellular component.

The selected training gene set for the complete gene expression-based model was found to be enriched in cellular components related to plasma membrane, endoplasmic-reticulum membrane, organelle membrane and nuclear-endoplasmic reticulum membrane (Fig. 8a). Mitochondria-associated ER-membrane responds to various stress signals including apoptotic signalling, inflammatory signalling and unfolded protein response (UPR). These pathways may be perturbed due to abnormal or uncontrolled expression of related genes resulting in cancer development^68^. Training Gene set for the driver gene expression-based model is enriched in cellular component such as plasma, membrane and organelle membrane (Fig. 8b).

**Figure 9:**
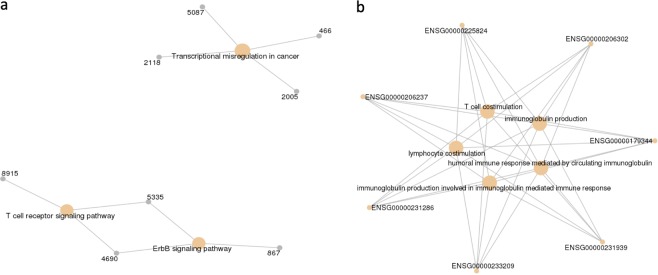
(**a**) Gene ontology analysis of gene set from driver gene expression-based model for biological process. (**b**) Gene ontology analysis of gene set by complete gene expression-based model for biological process.

Gene set from driver gene-based model is enriched in biological processes related to transcriptional misregulation and ErbB signaling (Fig. 9a). Transcription factors are involved in tumorigenesis by altering expression profiles of their targets^69^. ErbB tyrosine kinase receptors are found to be activated by epidermal growth factor controlling cellular proliferation, angiogenesis and metastasis in breast cancer^70^. Gene features from the complete Gene expression-based models are more enriched in biological process related to immunological response such as T cell costimulation, immunoglobin response (Fig. 9b). Impaired expression of HLA-DQB1 due to change in methylation pattern of gene is associated with esophageal squamous cell carcinoma by altering immune response pattern^71^.

## Methods

### Data mining

The study dataset was obtained from TCGA using TCGA2STAT R package, which automatically downloads and processes TCGA genomics and clinical data into a format convenient for statistical analyses in R environment^27^. The package imports and processes molecular profile from high-throughput experiments such as microarray, next generation sequencing and methylation array.

### Data pre-processing and normalization

As an initial step of pre-processing, which aids in preliminary feature reduction for a feature-rich training dataset, gene features showing near zero variance across the two classes were removed. Near zero variance features are the feature which either have unique value or have few unique value relative to the number of samples. Along similar lines, the features having more than 80% correlation with each other can prove to be problematic for machine-learning. Hence, such feature pairs/groups were also removed in a way where only a single feature of the group remains. These two tasks were performed using the Caret, a R package^28^. We used RPKM (Reads Per Kilobase of transcript per Million mapped reads) values of the reads for supervised machine-learning analysis. RPKM is a measure of normalization of RNA-seq data with the total read length and number of sequencing reads for a given sample^72^. The training datasets were standardized using z-score normalization. The normalization converts all the features to common scale with mean zero and standard deviation 1. The normalized data-set was used for training models.

### Feature selection

Feature selection is an advantageous step before machine-learning which reduces the dimensionality of datasets^26^. Given the possibly large sets of features, it helps in searching for the subset of features that has relevance in terms of a given predictor variable^73^. It also helps in improving the accuracy of a classifier by removing irrelevant data^74^. The main challenge associated with current data mining technologies is the high dimensionality of datasets combined with homogenous nature of data^75^.

For reducing the dimensionality of the datasets and identifying relevant features for building efficient machine-learning classifiers, we implemented various feature selection algorithms such as RFE, RLASSO, Random forest, linear modelling and linear regression, which provide individual ranking to gene features. Recursive Feature Extraction (RFE) is a method which utilizes recursion for feature extraction where smaller and smaller sets are considered as features until the desired number of features are returned. Randomized lasso is a stability selection method, which is combination of sub-sampling of high dimensional datasets and selection algorithm^76^. Linear regression assumes that features which are important have highest coefficient in the model, and features which have low importance have lower coefficient in the model. When there are multiple correlated features, small change in data can lead to large change in model. Regression model uses regularization method which adds an additional penalty to a model in order to minimize the sum of squared error of training model using lasso and ridge regression methods. Lasso regression methods performs L1 regularization minimizes absolute sum of the coefficient and producing sparse solution. Ridge regression performs L2 regularization minimizing squared absolute sum of the coefficients. The Least absolute shrinkage and selection operator (LASSO) does regression analysis for parameter estimation and variable selection simultaneously^77^. Random forest uses decision tree based strategies to rank feature based on attribute “feature importance”. All of the feature selection methods were implemented using the Python 3.6 scikit-learn library.

These feature-selection methods were used to rank the gene features of the training datasets. All the methods were implemented using the Python 3.6 scikit-learn library. All of the above-mentioned methods report individual ranking for the features. In order to get consensus ranking, we calculated the overall mean of each feature rank obtained from individual method. Subsequently, the Top 20, 30, 40, 50, 60 and 80 features were used to train and evaluate accuracy of models for binary classification of early versus late IDC, based on 5 machine-learning methods namely - RF, Naive Bayes, SVM, Logistic regression (LR) and Decision tree. Gene features list which gave the highest accuracy for all the machine-learning method were selected for model generation and evaluation. t-SNE technique was used for visualization of our gene expression datasets, returned after feature selection, in order to check if data-sets are segregating into defined class based on selected features for visualization of high dimensional data-point. t-SNE uses random walk on neighbourhood graph that allows implicit structure of data point to influence the way groups of data is present^78^.

### Handling data imbalance

Real world datasets are imbalanced, predominately composed of “normal” example and a small percentage of “abnormal” examples^79^. Imbalance results in poor predictive accuracy of minority class and difficulty in assessment of performance of classifier as most new sample are classified into minority class^80^. Class imbalanced datasets shows biasness which can be attenuated using SMOTE resulting in class balanced datasets^80^. Feature space similarity between minority classes are used to generate artificial data in SMOTE resampling^81^. It has been proven that over sampling of minority class with under sampling of majority class results in improvement of accuracy^79^. This method has been used to increase predicative accuracy of model for multiclass microarray datasets^82^. To check the usage of SMOTE resampling, models were trained on datasets where SMOTE resampling was employed.

### Training classification models

After feature selection and data processing, we trained different algorithms to generate efficient classifiers for early and late tumour stage. We used five different algorithms – Random Forest, Naive Bayes, LinSVM (Support Vector Machines with linear kernels), Logistic regression and Decision tree. Naive Bayes is based on Bayesian theorem that calculates the probability of attribute to fall in particular instance with the assumption that every attribute is independent from other attributes^83^. Random forest uses ensemble of decision tree by random selection of features to split node^84^. SVM implements Sequential Optimization Algorithm for decision function^85^.

### Training-cum-validation

The five supervised machine-learning algorithms (Random Forest, Naive Bayes, LinSVM, Logistic regression and Decision tree) were trained on subset of features obtained from feature selection and validated by 10-fold cross validation. The training models were compared by their accuracy, auROC, precision -recall and F-measure value.

### Independent data testing

We further re-evaluated the best-trained model on an independent dataset which was not used in the classifier training at all.

### Calculating threshold expression values for selected gene features

We performed differential expression analysis for the selected gene features by the two models, for early-late datasets to find out the differential expression of gene features selected by our model. Each gene feature selected by our model had range of expression across all the samples. We executed machine-learning and model evaluation for every single feature selected by our classifiers with threshold set across its expression range. The value that was giving highest ROC was considered as threshold value of expression value that could discriminate between early-late stages. Threshold value is the expression value beyond which the sample will segregate into two groups, in our case ‘early’ and ‘late’ stage.

### Cancer driver gene expression-based model

The available driver gene list for the cancer were also used for building model to discriminate early-late stages of breast cancer. We compiled a list of driver genes using Cosmic, intoGen and baile, which are expert curated lists of driver genes in human cancers. Cosmic stands for catalogue of somatic mutations in cancer which is an expert curated list of driver genes in human cancers, which is widely used in medical research^29^. IntoGen identifies somatic mutation, gene, pathways that are involved in tumorigenesis by analysis of 13 cancers^30^. Bailey list identifies 299 molecular cancer genes by pan-cancer and pan-software analysis of 9,423 tumour exome datasets using 26 computational tools^31^. We reduced the data-set to these gene features, which was then used for feature selection and model building, repeating the above-mentioned steps to generate driver gene expression-based model for the web server.

### Gene ontology

GO was performed on the list of genes returned by the feature selection methods to determine which gene families play role in the progression of breast cancer. We performed enrichment analysis using clusterprofiler R package. The package makes use of the datasets from the post genomic era high throughput technologies such as RNA-seq, micro-array, etc. to examine cellular molecules at systems level^86^. We also performed STRING protein-protein interaction analysis to discover major pathways targeted by selected gene features.

### External data-set evaluation

To further check the performance of our model, we obtained independent datasets form GEO with accession ID GSE61304 containing 60 samples of IDC with clinical stage information obtained using microarray profiling. GEOquery package helps the user to access the information stored in GEO directly using Bioconductor without any formatting or parsing problem^87^. Biomart was used to annotate the probe IDs of microarray datasets with gene symbols^88^. If a particular probe is sequenced multiple times, WGCNA R package collapserow function was used to select one single representative row of each probe ID^89^. Subsequently, RMA normalization was performed using GCRMA R package converting the expression in log 2 scale to make its distribution comparable to RNA-seq datasets^90,91^. This independent-testing dataset were segregated into driver-testing datasets and complete gene expression datasets for performance evaluation of the generated models.

## Conclusion

We have successfully applied supervised machine-learning classification on gene expression profiles to develop classification models for discriminating early and late stage of invasive ductal carcinoma. The RNA-seq data obtained from TCGA had various additional information related to the samples ranging from age, survivability, TNM staging, histological subtype and pathological stage in the form of metadata or clinical data.

The data yielded 20,505 gene expression values used as training features to be considered for classification model trainings. This voluminous dimensionality was reduced using various data pre-processing and feature selection methods. After this, the classifier models were generated by applying various machine-learning algorithms. Based on best trained classifiers, we developed a web-server Duct-BRCA-CSP that could predict whether the sample represents early or late stage using patient’s gene expression profile. The expression-based gene features returned by the feature selection methods can be used to differentiate samples between early and late stage with high accuracy, also providing candidate biomarkers for improved diagnosis and treatment, subsequent to adequate experimental validations. Thus, the combined power of machine-learning and next generation sequencing can provide important insights into the progression IDC from early to late stage. Our study proves that accurate prediction models captured features of the relevant genes, that could be candidates for further experimental evaluation for therapeutic and prognostic potential in the cancer treatment.

## Discussion

We developed a web-server Duct-BRCA-CSP for invasive ductal carcinoma which predicts tumour stage of a sample on the basis of RNA-seq expression profile of selected genes, rather than its tumour size, gene expression profile of all the genes, imaging or survivability. Our study is preliminary in nature, however, in the future, the availability of datasets from higher number of patients, especially those representing late stage may help in building more efficient stage specific classifiers which may be suitable for personalized treatment strategy in clinics. In addition, further inclusion of more datasets such as mutation profile, methylation data and protein isoform data may improve accuracy of classifiers. Inclusion of paired datasets can also further aid in gaining valuable insights into the progression of breast cancer. To the best of our knowledge, the webserver Duct-BRCA-CSP is a server which is first of its kind for prediction of IDC tumour stages based on gene expression profiles of selected genes.

## Supplementary information

Supplementary information.
