# Supplementary material for: Classification models for Invasive Ductal Carcinoma Progression, based on gene expression data-trained supervised machine learning
Source: Sci Rep. 2020 Mar 5;10:4113. doi: 10.1038/s41598-020-60740-w (PMC7057992; doi:10.1038/s41598-020-60740-w)
Supplement: Supplementary file 1 — Supplementary information. [file 41598_2020_60740_MOESM1_ESM.pdf]

Classification models for Invasive Ductal Carcinoma Progression, based on gene expression data-trained supervised machine learning

Shikha Roy, Rakesh Kumar, Vaibhav Mittal, Dinesh Gupta*

International Centre for Genetic Engineering and Biotechnology, New Delhi, India

***Corresponding Author –**

Phone: +91 26743007

Fax: +91 26742316

Email – dinesh@icgeb.res.in (DG)

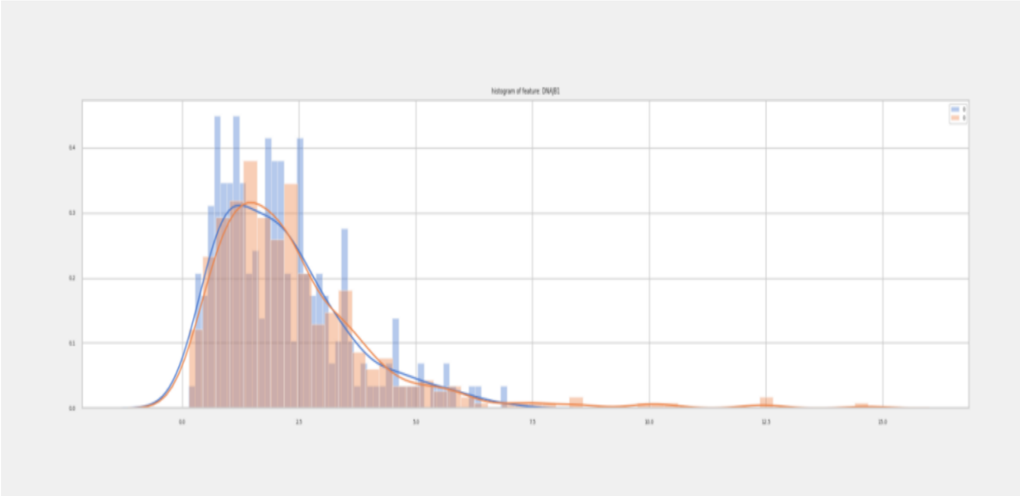

Figure S1: Distribution plot of gene DNAJB1 expression before normalization

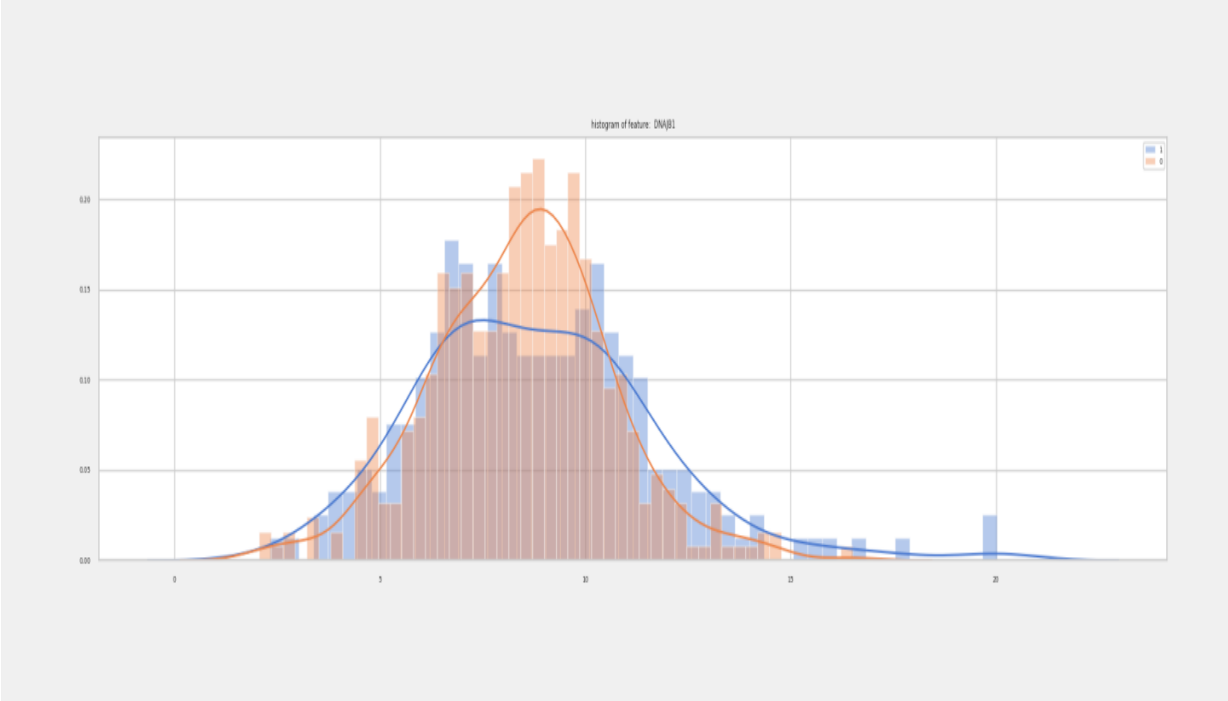

Figure S2: Distribution plot of gene DNAJB1 expression after normalization

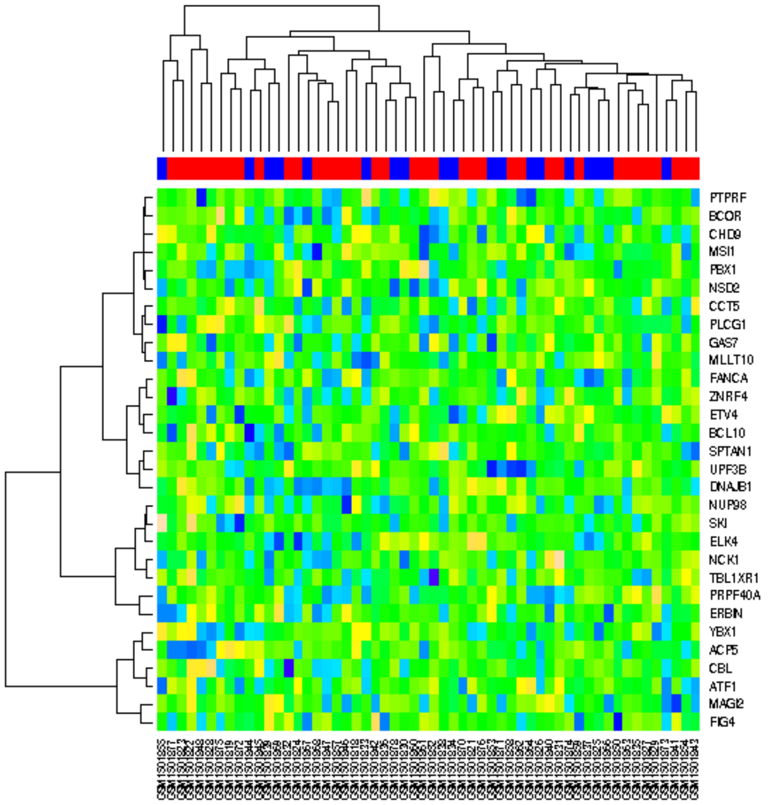

Figure S3: Heatmap of differential expression between early and late IDC stages for the genes set from complete gene expression-based model.
Red: Early stage Blue: Late stage

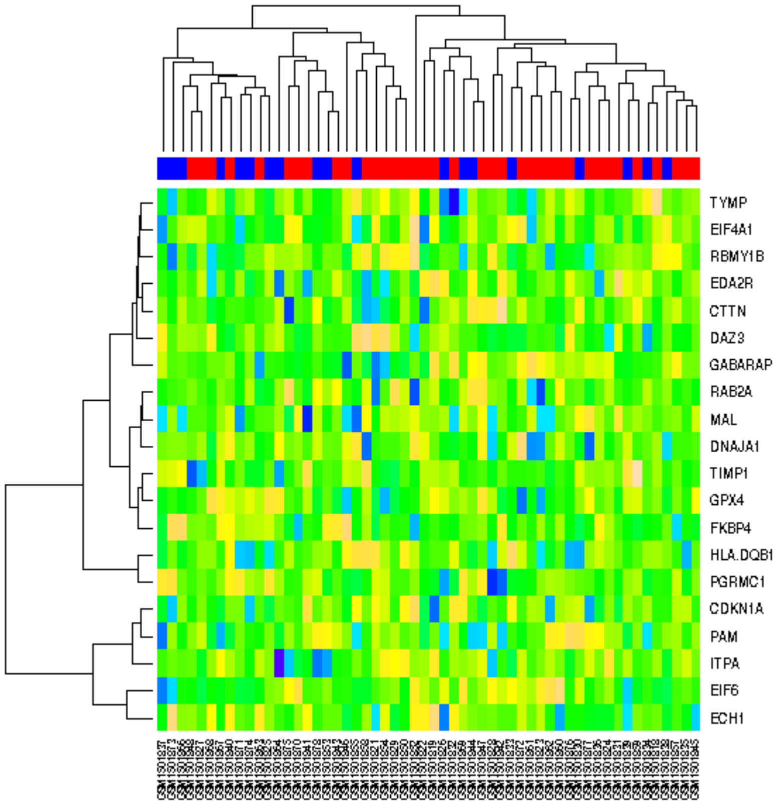

Figure S4: Heatmap of differential expression between early and late IDC stages for gene set from driver gene expression-based model.
Red: Early stage Blue: Late stage

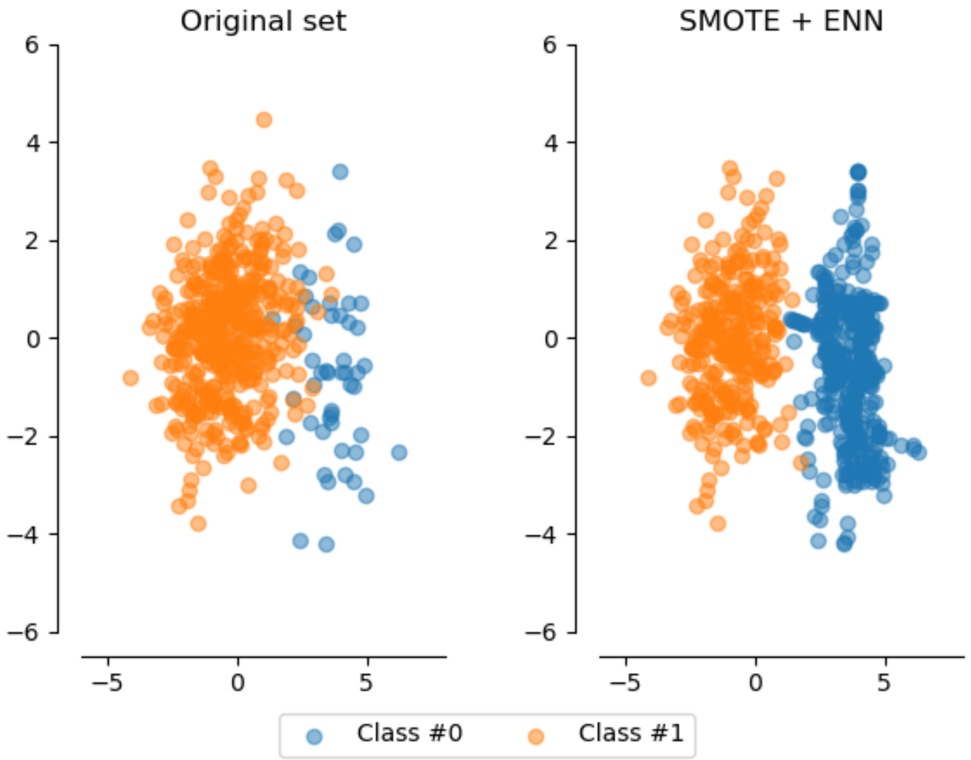

Figure S5: Due to high class imbalance (461 early stage versus 161 late stage) Synthetic Minority Oversampling technique (SMOTE) was employed used python scikit-learn.   Scatter plot to evaluate effectiveness of SMOTE + ENN re-sampling technique to handle class imbalance. Early stage datasets are labelled as #1, and late stage labelled as #0. a) Prior to resampling b.) Post SMOTE resampling. As compared to original sample prior to resampling, post SMOTE resampling the more late stage samples are generated using k- neighbour of majority class.

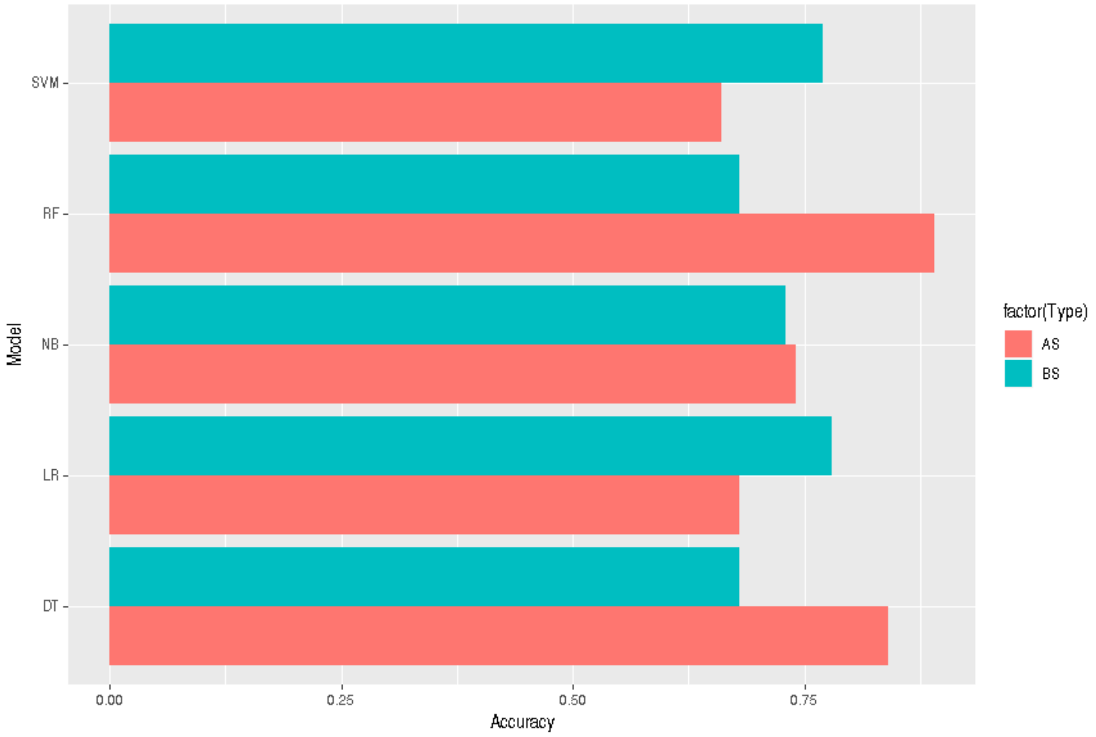

Figure S6: SMOTE resampled datasets were used to train the binary classification model and their accuracy was again evaluated. Majority of algorithm was showing improved accuracy of classification model after SMOTE resampling. Accuracy of machine learning algorithm before SMOTE resampling and after SMOTE resampling. NB: Naïve Bayes, LR: Logistic Regression, RF: Random Forest, SVM: Support Vector Machine, DT: Decision Tree.BS: Before SMOTE resampling AS: After SMOTE resampling. X axis: Machine learning algorithm Y axis: Accuracy.

Survival curve for each model

Overall Survival Kaplan-Meier Estimate

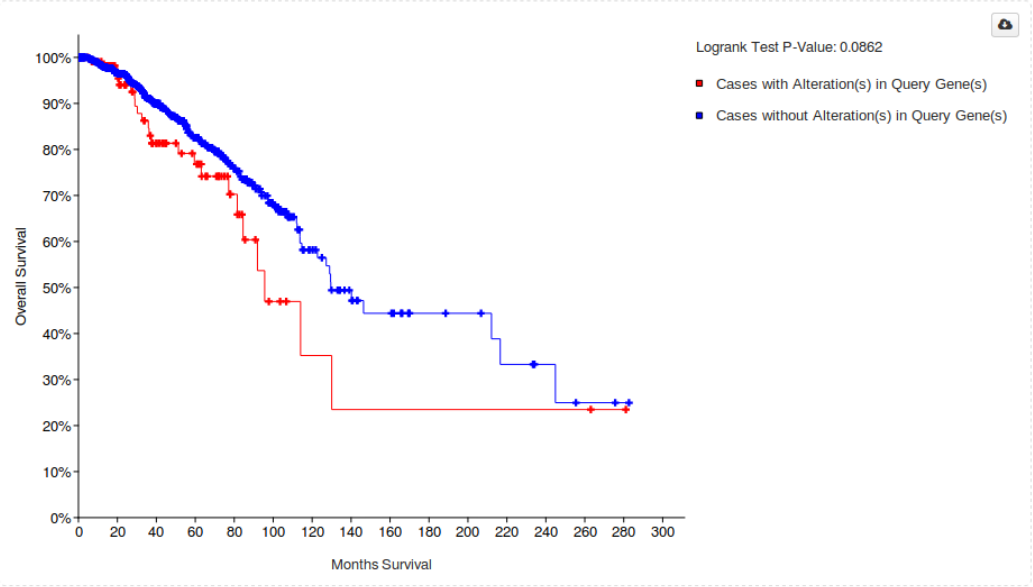

	Number of Cases, Total	Number of Cases, Deceased	Median Months Survival
Cases with Alteration(s) in Query Gene(s)	**124**	**23**	**95.63**
Cases without Alteration(s) in Query Gene(s)	**862**	**120**	**129.6**

Figure S7: Survival curve for gene set from driver gene expression-based model.

`

Overall Survival Kaplan-Meier Estimate

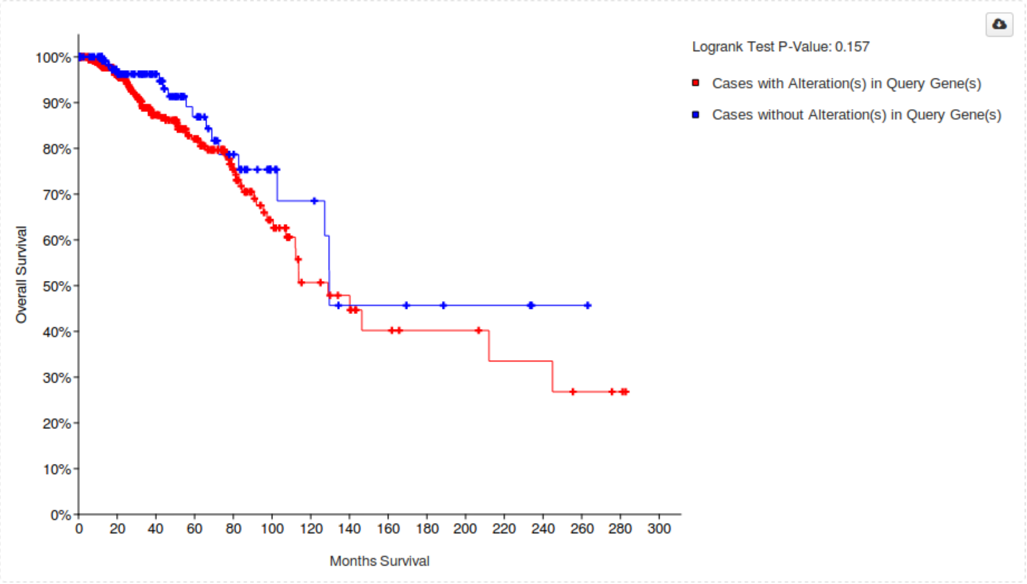

	Number of Cases, Total	Number of Cases, Deceased	Median Months Survival
Cases with Alteration(s) in Query Gene(s)	**395**	**66**	**128.98**
Cases without Alteration(s) in Query Gene(s)	**131**	**17**	**129.6**

Figure S8: Survival curve for gene set from complete gene-expression based gene model.

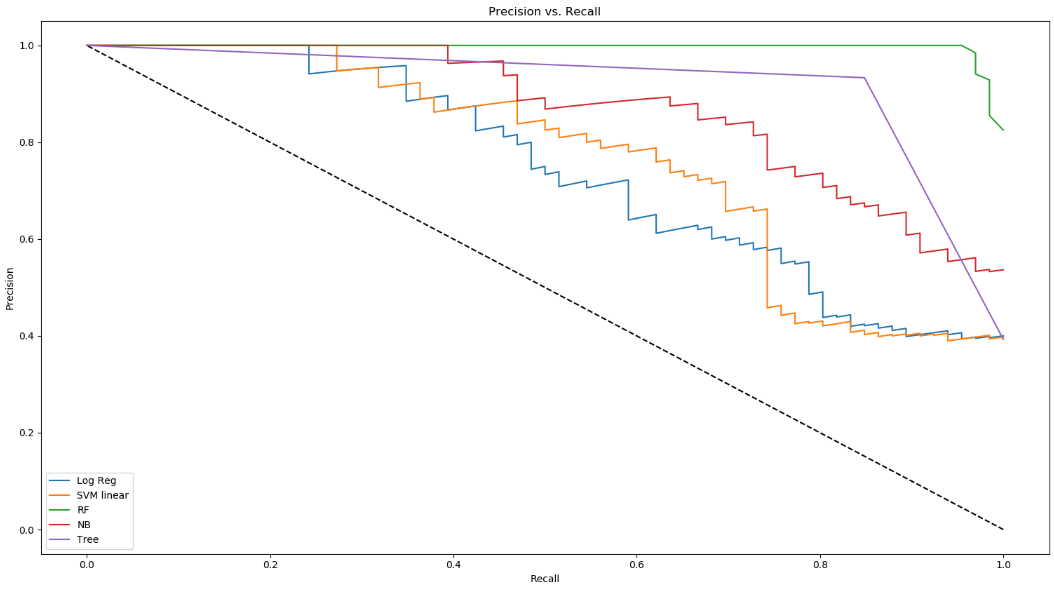

Figure S9: AUPRC of driver gene expression-based model from testing datasets. Amongst all the prediction models, Random Forest achieved the maximum area under precision-recall curve for driver-gene expression-model.

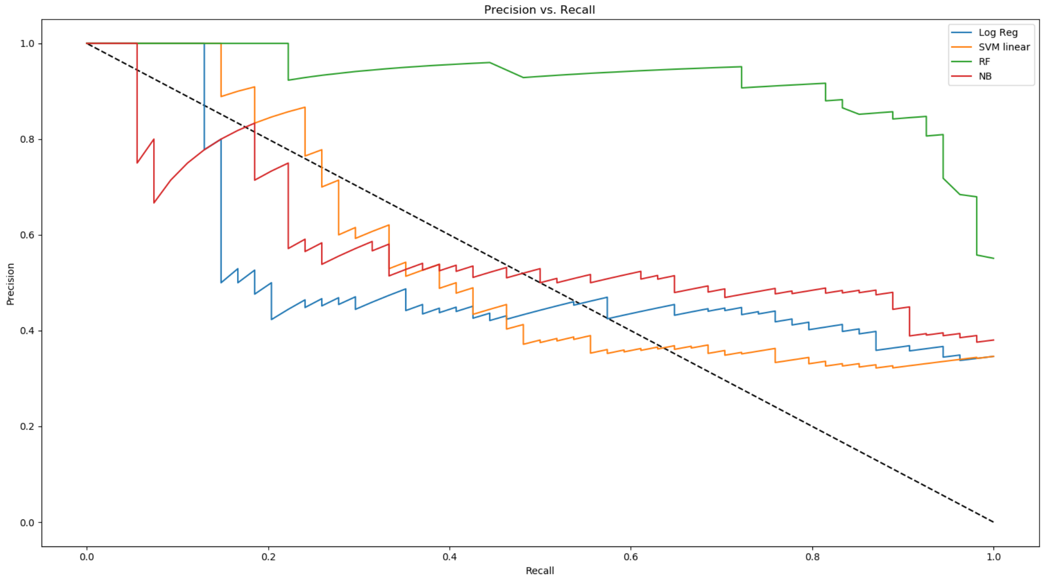

Figure S10: AUPRC of complete gene expression based-model from testing datasets. Amongst all the prediction models, Random Forest achieved the maximum area under precision-recall curve for driver-gene expression-model.

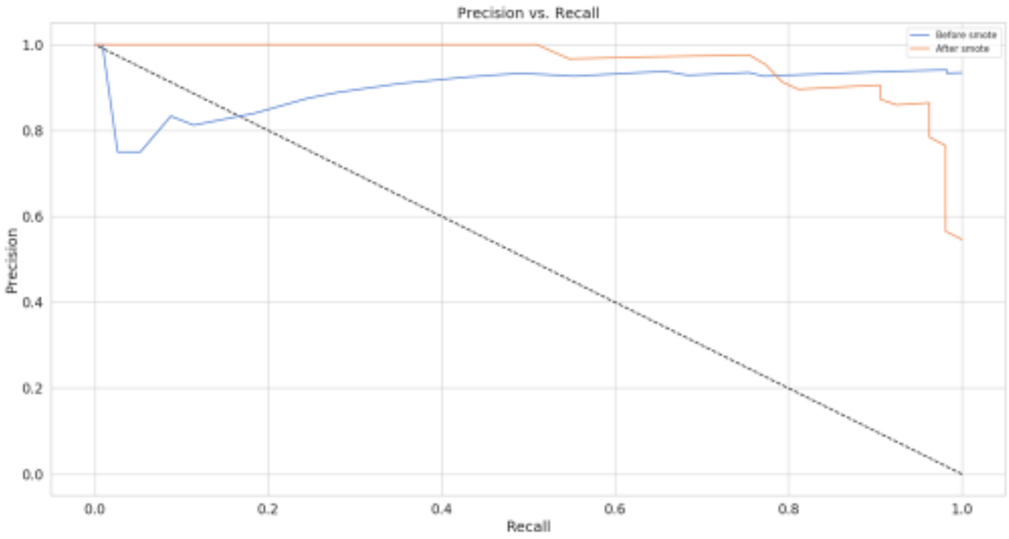

Figure S11: AUPRC before and after SMOTE resampling of driver expression based-model for testing datasets.

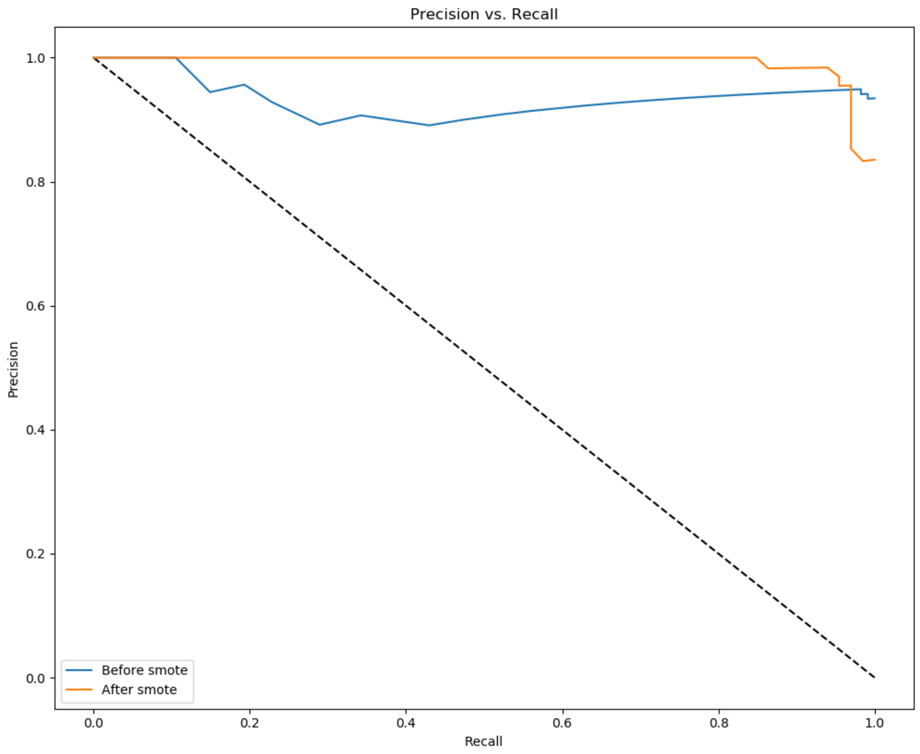

Figure S12: AUPRC before and after SMOTE resampling of driver gene expression based-model for external datasets.

Supplementary Table ST1: Gene symbols and gene name and literature validation of selected gene features. It consists of 50 gene selected by complete gene expression-based model and driver gene expression-based model.

S.No.	Gene Symbol	Gene Name	Associated Cancer Type	Remarks and References
1.	DNAJB1		Breast carcinoma, hepatocellular carcinoma	It negatively regulates MIG6, which is a tumor suppressor resulting in cancer cell proliferation by EGFR signaling pathway. [1]
2.	GAS7		Breast cancer	It is directly regulated by p53, which results in suppression of metastasis in breast cancer. [2]
3.	BCOR		T-cell lymphoblastic leukemia	Functions as a tumor suppressor and its mutation has been identified to be associated with various hematological malignancies.[3]
4.	SKI		Breast cancer	Its divergent expression is associated with fibroblast in tumor microenvironment in breast cancer. [4]
5.	ETV4		Breast cancer	Its overexpression is associated with distant metastasis and poor prognosis in breast cancer. [5]
6.	MLLT10		T- acute lymphoblastic leukemia	MLLT10 has specific leukemia fusion with PICALM in T- acute lymphoblastic leukemia.[6]
7.	UPF3B		--	--
8.	CBL		Breast cancer	It functions as adaptor protein with E3 ubiquitin ligase and its mutation is associated with carcinogenesis.[7]
9.	PBX1		Breast Cancer	Its altered expression is associated with ER meditated transcriptional response in driving breast cancer. [8]
10.	ELK4		Breast Cancer	It is an ETS transcription factor whose copy number changes is associated with pathogenesis of cancer.[9]
11.	NCK1		Breast cancer, Lung cancer	Regulates breast cancer prognosis and metastasis by growth and vascularization of primary tumor.[10]
12.	PTPRF		Cancer	It is an important cell cycle regulator associated with tyrosine kinase which regulates cell proliferation, apoptosis, migration and invasion.[11]
13.	ATF1		Breast cancer	It belongs to a family of transcription factor that regulates cell cycle and apoptosis in cancer progression.[12]

14.	MAGI2	Breast cancer	It is a tumor suppressor, which is down-regulated in breast cancer.[13]
15.	ERBB2IP	Breast cancer	It belongs to a family of receptor tyrosine kinase which regulates breast tumor formation and progression.[14]
16.	SPTAN1	OsteoSarcoma	(Gene Cards)
17.	FANCA	Breast cancer	FANCA gene duplication is associated with increased risk of breast cancer.[15]
18.	TRRAP	Ovarian cancer	Its over-expression is associated with increased proliferation and stemness of ovarian cancer.[16]
19.	CHD9	Breast cancer	It belongs to a family of chromatin regulator whose inactivation in cancer results in distortion to cellular machinery related to transcription and DNA damage response.[17]
20.	WHSC1	Cervical cancer	Its hypomethylation results in over-expression that promotes cervical carcinogenesis.[18]
21.	GOPC	Colorectal cancer	It's down-regulation is associated with venous invasion and poor prognosis in colorectal cancer.[19]
22.	PRPF40B	Acute myeloid leukemia	It is a spliceosome complex gene which is mutated in acute myeloid leukemia.[20]
23.	NUP98	Murine carcinoma, Hepatocellular carcinoma	Regulates the expression of p21 and its down-regulation is associated with hepatocellular carcinoma.[21]
24.	CCT5	Breast cancer	It implicates resistance to docetaxel treatment in breast cancer.[22]
25.	PLCG1	Cancer	It is a phospholipase enzyme that promotes cell invasion, metastasis and tumor progression in cancer.[23]
26.	BCL10	Ovarian cancer	It has role in DNA damage response and associated with tumor aggression and poorer prognosis.[24]
27.	ZNRF3	--	
28.	YBX1	Breast Cancer	It is a transcription and translation related protein over-expressed in various malignancies.[25]
29.	MSI2	Breast cancer	It is upstream regulator of ESR1 which modulates estrogen receptor pathway that results in cancer cell growth.[26]
30.	TBL1XR1	Gastric cancer	It is over-expressed in gastric cancer and can serve as therapeutic target.[27]

31.	CDKN1A	Breast cancer	Its over-expression correlates with tumor size and lymph node metastasis in breast cancer.[28]
32.	FKBP4	--	--
33.	DAZ3	Breast cancer	
34.	DNAJA1	Breast Cancer	It controls mutant p53 which contributes to malignancy in breast cancer.[29]
35.	ECH1	Breast Cancer	Its over-expression is associated with increased adipogenesis in breast cancer cell.[30]
36.	RBMY1B	Breast cancer	--
37.	GABARAP	Breast Cancer	It is associated with lymph node metastasis in breast cancer. [31]
38.	MAL2	Ovarian cancer, Cervical cancer	Its down-regulation results in redistribution of lipid raft in cancer. [32]
39.	EDA2R	Breast cancer	Its transcriptional activation results in cell death in breast cancer.[33]
40.	ITPA	Cancer	It encodes enzyme that is responsible for deamination of adenine that results in mutagenesis and DNA damage response.[34]
41.	EIF4A1	Breast cancer	It encodes protein that is involved in dys-regulation of protein synthesis that results in tumorigenesis.[35]
42.	CTTN	Melonoma, Breast cancer, Colorectal cancer	It encodes cortactin-regulator of actin cytoskeletal which is having amplification in breast cancer. [36]
43.	GPX4		
44.	RAB18	Breast cancer	It dys-regulation results in breast cancer proliferation and invasion.[37]
45.	EIF6	Colorectal cancer, Head and neck carcinoma	Its overexpression results in activation of Wnt signaling pathway that regulates cell division and promotes oncogenesis.[38]
46.	TIMP1	Breast cancer	It encodes protein which directly regulates apoptosis and metastasis in triple negative breast cancer.[39]
47.	HLA-DQB1	Breast cancer	It is found to have protective role in immune surveillance process of breast cancer.[40]
48.	TYMP	Rectal cancer	It is over-expressed in cancer and promotes process related to angiogenesis.[41]
49.	PAM	--	--
50.	PGRMC1	Breast cancer, Ovarian cancer	It is found to have mutually exclusive expression with ER in breast cancer.[42]

1. Park SY, Choi HK, Seo JS, Yoo JY, Jeong JW, Choi Y, Choi KC, Yoon HG: **DNAJB1 negatively regulates MIG6 to promote epidermal growth factor receptor signaling**. *Biochimica et biophysica acta* 2015, **1853**(10 Pt A):2722-2730.
2. Chang JW, Kuo WH, Lin CM, Chen WL, Chan SH, Chiu MF, Chang IS, Jiang SS, Tsai FY, Chen CH *et al*: **Wild-type p53 upregulates an early onset breast cancer-associated gene GAS7 to suppress metastasis via GAS7-CYFIP1-mediated signaling pathway**. *Oncogene* 2018, **37**(30):4137-4150.
3. Tanaka T, Nakajima-Takagi Y, Aoyama K, Tara S, Oshima M, Saraya A, Koide S, Si S, Manabe I, Sanada M *et al*: **Internal deletion of BCOR reveals a tumor suppressor function for BCOR in T lymphocyte malignancies**. *The Journal of experimental medicine* 2017, **214**(10):2901-2913.
4. Wang L, Hou Y, Sun Y, Zhao L, Tang X, Hu P, Yang J, Zeng Z, Yang G, Cui X *et al*: **c-Ski activates cancer-associated fibroblasts to regulate breast cancer cell invasion**. *Molecular oncology* 2013, **7**(6):1116-1128.
5. Dumortier M, Ladam F, Damour I, Vacher S, Bieche I, Marchand N, de Launoit Y, Tulasne D, Chotteau-Lelievre A: **ETV4 transcription factor and MMP13 metalloprotease are interplaying actors of breast tumorigenesis**. *Breast cancer research : BCR* 2018, **20**(1):73.
6. Brandimarte L, Pierini V, Di Giacomo D, Borga C, Nozza F, Gorello P, Giordan M, Cazzaniga G, Te Kronnie G, La Starza R *et al*: **New MLLT10 gene recombinations in pediatric T-acute lymphoblastic leukemia**. *Blood* 2013, **121**(25):5064-5067.
7. Kang JM, Park S, Kim SJ, Hong HY, Jeong J, Kim HS, Kim SJ: **CBL enhances breast tumor formation by inhibiting tumor suppressive activity of TGF-beta signaling**. *Oncogene* 2012, **31**(50):5123-5131.
8. Magnani L, Ballantyne EB, Zhang X, Lupien M: **PBX1 genomic pioneer function drives ERalpha signaling underlying progression in breast cancer**. *PLoS genetics* 2011, **7**(11):e1002368.
9. Mesquita B, Lopes P, Rodrigues A, Pereira D, Afonso M, Leal C, Henrique R, Lind GE, Jeronimo C, Lothe RA *et al*: **Frequent copy number gains at 1q21 and 1q32 are associated with overexpression of the ETS transcription factors ETV3 and ELF3 in breast cancer irrespective of molecular subtypes**. *Breast cancer research and treatment* 2013, **138**(1):37-45.
10. Morris DC, Popp JL, Tang LK, Gibbs HC, Schmitt E, Chaki SP, Bywaters BC, Yeh AT, Porter WW, Burghardt RC *et al*: **Nck deficiency is associated with delayed breast carcinoma progression and reduced metastasis**. *Molecular biology of the cell* 2017, **28**(24):3500-3516.
11. Du Y, Grandis JR: **Receptor-type protein tyrosine phosphatases in cancer**. *Chinese journal of cancer* 2015, **34**(2):61-69.
12. Thompson MR, Xu D, Williams BR: **ATF3 transcription factor and its emerging roles in immunity and cancer**. *Journal of molecular medicine* 2009, **87**(11):1053-1060.
13. Yang Y, Yang H, Xu M, Zhang H, Sun M, Mu P, Dong T, Du S, Liu K: **Long non-coding RNA (lncRNA) MAGI2-AS3 inhibits breast cancer cell growth by targeting the Fas/FasL signalling pathway**. *Human cell* 2018, **31**(3):232-241.
14. Tao Y, Shen C, Luo S, Traore W, Marchetto S, Santoni MJ, Xu L, Wu B, Shi C, Mei J *et al*: **Role of Erbin in ErbB2-dependent breast tumor growth**. *Proceedings of the National Academy of Sciences of the United States of America* 2014, **111**(42):E4429-4438.
15. Abbasi S, Rasouli M: **A rare FANCA gene variation as a breast cancer susceptibility allele in an Iranian population**. *Molecular medicine reports* 2017, **15**(6):3983-3988.
16. Kang KT, Kwon YW, Kim DK, Lee SI, Kim KH, Suh DS, Kim JH: **TRRAP stimulates the tumorigenic potential of ovarian cancer stem cells**. *BMB reports* 2018, **51**(10):514-519.
17. Mills AA: **The Chromodomain Helicase DNA-Binding Chromatin Remodelers: Family Traits that Protect from and Promote Cancer**. *Cold Spring Harbor perspectives in medicine* 2017, **7**(4).

18. Yin Z, Sun Y, Ge S, Sun J: **Epigenetic activation of WHSC1 functions as an oncogene and is associated with poor prognosis in cervical cancer**. *Oncology reports* 2017, **37**(4):2286-2294.
19. Ohara N, Haraguchi N, Koseki J, Nishizawa Y, Kawai K, Takahashi H, Nishimura J, Hata T, Mizushima T, Yamamoto H *et al*: **Low expression of the GOPC is a poor prognostic marker in colorectal cancer**. *Oncology letters* 2017, **14**(4):4483-4490.
20. Watson IR, Takahashi K, Futreal PA, Chin L: **Emerging patterns of somatic mutations in cancer**. *Nature reviews Genetics* 2013, **14**(10):703-718.
21. Singer S, Zhao R, Barsotti AM, Ouwehand A, Fazollahi M, Coutavas E, Breuhahn K, Neumann O, Longerich T, Pusterla T *et al*: **Nuclear pore component Nup98 is a potential tumor suppressor and regulates posttranscriptional expression of select p53 target genes**. *Molecular cell* 2012, **48**(5):799-810.
22. Ooe A, Kato K, Noguchi S: **Possible involvement of CCT5, RGS3, and YKT6 genes up-regulated in p53-mutated tumors in resistance to docetaxel in human breast cancers**. *Breast cancer research and treatment* 2007, **101**(3):305-315.
23. Sala G, Dituri F, Raimondi C, Previdi S, Maffucci T, Mazzoletti M, Rossi C, Iezzi M, Lattanzio R, Piantelli M *et al*: **Phospholipase Cgamma1 is required for metastasis development and progression**. *Cancer research* 2008, **68**(24):10187-10196.
24. Ismail IH, Dronyk A, Hu X, Hendzel MJ, Shaw AR: **BCL10 is recruited to sites of DNA damage to facilitate DNA double-strand break repair**. *Cell cycle* 2016, **15**(1):84-94.
25. Lim JP, Shyamasundar S, Gunaratne J, Scully OJ, Matsumoto K, Bay BH: **YBX1 gene silencing inhibits migratory and invasive potential via CORO1C in breast cancer in vitro**. *BMC cancer* 2017, **17**(1):201.
26. Kang MH, Jeong KJ, Kim WY, Lee HJ, Gong G, Suh N, Gyorffy B, Kim S, Jeong SY, Mills GB *et al*: **Musashi RNA-binding protein 2 regulates estrogen receptor 1 function in breast cancer**. *Oncogene* 2017, **36**(12):1745-1752.
27. Liu F, He Y, Cao Q, Liu N, Zhang W: **TBL1XR1 Is Highly Expressed in Gastric Cancer and Predicts Poor Prognosis**. *Disease markers* 2016, **2016**:2436518.
28. Wei CY, Tan QX, Zhu X, Qin QH, Zhu FB, Mo QG, Yang WP: **Expression of CDKN1A/p21 and TGFBR2 in breast cancer and their prognostic significance**. *International journal of clinical and experimental pathology* 2015, **8**(11):14619-14629.
29. Parrales A, Ranjan A, Iyer SV, Padhye S, Weir SJ, Roy A, Iwakuma T: **DNAJA1 controls the fate of misfolded mutant p53 through the mevalonate pathway**. *Nature cell biology* 2016, **18**(11):1233-1243.
30. Johnstone CN, Mongroo PS, Rich AS, Schupp M, Bowser MJ, Delemos AS, Tobias JW, Liu Y, Hannigan GE, Rustgi AK: **Parvin-beta inhibits breast cancer tumorigenicity and promotes CDK9-mediated peroxisome proliferator-activated receptor gamma 1 phosphorylation**. *Molecular and cellular biology* 2008, **28**(2):687-704.
31. Hervouet E, Claude-Taupin A, Gauthier T, Perez V, Fraichard A, Adami P, Despouy G, Monnien F, Algros MP, Jouvenot M *et al*: **The autophagy GABARAPL1 gene is epigenetically regulated in breast cancer models**. *BMC cancer* 2015, **15**:729.
32. Horne HN, Lee PS, Murphy SK, Alonso MA, Olson JA, Jr., Marks JR: **Inactivation of the MAL gene in breast cancer is a common event that predicts benefit from adjuvant chemotherapy**. *Molecular cancer research : MCR* 2009, **7**(2):199-209.
33. Brosh R, Sarig R, Natan EB, Molchadsky A, Madar S, Bornstein C, Buganim Y, Shapira T, Goldfinger N, Paus R *et al*: **p53-dependent transcriptional regulation of EDA2R and its involvement in chemotherapy-induced hair loss**. *FEBS letters* 2010, **584**(11):2473-2477.
34. Ji D, Stepchenkova EI, Cui J, Menezes MR, Pavlov YI, Kool ET: **Measuring deaminated nucleotide surveillance enzyme ITPA activity with an ATP-releasing nucleotide chimera**. *Nucleic acids research* 2017, **45**(20):11515-11524.
35. Modelska A, Turro E, Russell R, Beaton J, Sbarrato T, Spriggs K, Miller J, Graf S, Provenzano E, Blows F *et al*: **The malignant phenotype in breast cancer is driven by eIF4A1-mediated changes in the translational landscape**. *Cell death & disease* 2015, **6**:e1603.
36. MacGrath SM, Koleske AJ: **Cortactin in cell migration and cancer at a glance**. *Journal of cell science* 2012, **125**(Pt 7):1621-1626.
37. Ye F, Tang H, Liu Q, Xie X, Wu M, Liu X, Chen B, Xie X: **miR-200b as a prognostic factor in breast cancer targets multiple members of RAB family**. *Journal of translational medicine* 2014, **12**:17.

38. Zhu W, Li GX, Chen HL, Liu XY: **The role of eukaryotic translation initiation factor 6 in tumors**. *Oncology letters* 2017, **14**(1):3-9.
39. Cheng G, Fan X, Hao M, Wang J, Zhou X, Sun X: **Higher levels of TIMP-1 expression are associated with a poor prognosis in triple-negative breast cancer**. *Molecular cancer* 2016, **15**(1):30.
40. Chaudhuri S, Cariappa A, Tang M, Bell D, Haber DA, Isselbacher KJ, Finkelstein D, Forcione D, Pillai S: **Genetic susceptibility to breast cancer: HLA DQB*03032 and HLA DRB1*11 may represent protective alleles**. *Proceedings of the National Academy of Sciences of the United States of America* 2000, **97**(21):11451-11454.
41. Derwinger K, Lindskog EB, Palmqvist E, Wettergren Y: **Changes in thymidine phosphorylase gene expression related to treatment of rectal cancer**. *Anticancer research* 2013, **33**(6):2447-2451.
42. Craven RJ: **PGRMC1: a new biomarker for the estrogen receptor in breast cancer**. *Breast cancer research : BCR* 2008, **10**(6):113.
